# An adaptive hybrid quadrature scheme: Combining Simpson’s rule and Gaussian quadrature for enhanced numerical integration

**DOI:** 10.1371/journal.pone.0335582

**Published:** 2026-02-23

**Authors:** Abadi Abraha Asgedom, Yohannes Yirga Kefela

**Affiliations:** Department of Mathematics, Mekelle University, Mekelle, Tigray, Ethiopia; Central State University, UNITED STATES OF AMERICA

## Abstract

This study develops a novel adaptive hybrid quadrature that combines Simpson’s 1/3 rule with Gauss-Legendre quadrature to overcome the classical difficulties in performing numerical integration. Classical methods may encounter challenges in achieving a good balance between computational cost and precision, especially when it comes to functions characterized by strongly varying behaviors across their domains. We address these issues via an intelligent adaptation mechanism that reallocates computing resources dynamically on localized function features. We rigorously analyse its convergence properties analytically and prove optimal error estimates in the sense of fourth order accuracy with a strong performance improvement. The hybrid error estimation methodology is based on the mathematical inconsistency of polynomial interpolation and orthogonal polynomial approximation which provides an effective device for local error evaluation. Extensive numerical results indicate that the proposed scheme is consistently better than several existing schemes with significant reduction in function evaluations and acceptable accuracy for different test functions. The proposed framework reduces computational costs by up to 62% when compared to traditional adaptive methods. It maintains similar precision. We carefully examine implementation details, complexity analysis, and practical deployment factors. This work is particularly relevant for scientific computing applications that require high-precision integration in computational physics, engineering simulations, and financial mathematics.

## 1 Introduction

Numerical integration remains fundamentally important throughout computational mathematics, with applications reaching into engineering, physics, finance, and data science [1,2]. The essential problem involves approximating definite integrals when analytical solutions prove unavailable. Classical Newton-Cotes formulas, such as Simpson’s rule, offer simplicity but limited efficiency for functions with localized complexity [3,4].

Gaussian quadrature methods, pioneered by Carl Friedrich Gauss, achieve exponential convergence for smooth functions through optimal node selection [5]. However, purely Gaussian approaches struggle with adaptive error estimation for functions containing singularities or rapid oscillations [6].

Adaptive quadrature strategies address these limitations by dynamically allocating computational resources. While contemporary methods have evolved from early Richardson-extrapolation approaches [7], many remain constrained by conservative error estimates that incur unnecessary overhead [8]. Recent hybrid frameworks, such as those combining Newton-Cotes and Gaussian rules [9], and global reconstruction techniques like mock-Chebyshev constrained least squares [10,11], demonstrate promising alternatives. Yet a key challenge persists: designing an efficient *switching criterion* that optimally selects between robust low-order and efficient high-order rules based on local integrand behavior.

This paper introduces an **Adaptive Hybrid Quadrature Scheme (AHQS)** that systematically combines Simpson’s 1/3 rule with Gauss-Legendre quadrature through a novel, cost-aware decision function. Our contributions are: (1) a mathematically defined hybrid error estimator and local efficiency index; (2) a clear adaptive algorithm (Algorithm 1) with rigorously justified parameters; (3) comprehensive numerical validation against standard adaptive routines and recent hybrid methods, demonstrating consistent reduction in function evaluations for integrands with localized irregularities.

## 2 Theoretical framework

### 2.1 Mathematical preliminaries

We begin by establishing the mathematical foundation for our hybrid approach. Consider the definite integral:

$$I=\int_{a}^{b}f(x)dx$$ (1)

where f:[a,b]→ℝ represents a sufficiently smooth function. Our methodology builds upon two classical quadrature techniques with complementary properties [4,5].

**Definition 2.1** (Composite Simpson’s 1/3 Rule). For an interval [*a*,*b*] partitioned into *n* equal subintervals (*n* even) with spacing h=(b−a)/n, the composite Simpson’s rule approximation becomes:

$$S(a,b)=\frac{h}{3}\left[f(a)+f(b)+4\sum_{k=1}^{n/2}f(x_{2k-1})+2\sum_{k=1}^{n/2-1}f(x_{2k})\right]$$ (2)

with error term

$$E_{S}=I-S(a,b)=-\frac{(b-a)h^{4}}{180}f^{\;2pt(4)}(\xi ),\;\xi \in [a,b]$$ (3)

provided $f\in C^{4}[a,b]$. This method exhibits fourth-order convergence and works particularly well for functions with moderate smoothness [1].

**Definition 2.2** (Gauss-Legendre Quadrature). The *m*-point Gauss-Legendre quadrature rule on [*a*,*b*] transforms to the standard interval [–1,1]:

$$G(a,b)=\frac{b-a}{2}\sum_{i=1}^{m}w_{i}f\left(\frac{b-a}{2}t_{i}+\frac{a+b}{2}\right)$$ (4)

where *t*_*i*_ denote roots of Legendre polynomial *P*_*m*_(*t*) and *w*_*i*_ represent corresponding weights:

$$w_{i}=\frac{2}{(1-t_{i}^{2})[P_{m}^{\prime }(t_{i}){]}^{2}}$$ (5)

For the 2-point rule used extensively here, $t_{1,2}=\pm \frac{1}{\sqrt{3}}$ with *w*_1,2_ = 1. The error term for 2-point Gauss-Legendre quadrature is:

$$E_{G}=I-G(a,b)=\frac{(b-a{)}^{5}}{4320}f^{\;2pt(4)}(\eta ),\;\eta \in [a,b]$$ (6)

This method achieves fourth-order accuracy with only two function evaluations per subinterval, representing optimal efficiency for polynomial exactness [2].

**Definition 2.3** (Hybrid Error Estimator). On a subinterval $I_{k}=[x_{k},x_{k+1}]$ of length *h*_*k*_, let *S*_*k*_(*f*) and *G*_*k*_(*f*) be the approximations from Definition 2.1 (with *n* = 2) and Definition 2.2 (with *m* = 2), respectively. The *hybrid error estimator* is defined as:


$$E_{\text{hybrid}}^{\;2pt(k)}:=|S_{k}(f)-G_{k}(f)|.$$

This estimator leverages the discrepancy between a low-order Newton–Cotes rule and a high-order Gaussian rule to locally gauge approximation error.

**Definition 2.4** (Local Efficiency Index). Let $I_{k}^{\text{true}}$ denote the true integral over *I*_*k*_. The *local efficiency index* of the hybrid estimator on *I*_*k*_ is defined as:


$$\eta_{k}:=\frac{E_{\text{hybrid}}^{\;2pt(k)}}{\left|I_{k}^{\text{true}}-G_{k}(f)\right|}.$$

An estimator is *efficient* if $\eta_{k}\to 1$ as $h_{k}\to 0$ for smooth *f*, and *reliable* if $\eta_{k}\geq 1$ for all *k*.

### 2.2 Hybrid error estimation theory

The core innovation of our approach lies in utilizing the discrepancy between Simpson’s rule and Gauss-Legendre quadrature as a robust error indicator. This cross-method comparison provides a more reliable estimate of local error than traditional approaches based on nested rules or Richardson extrapolation [12,13].

**Theorem 2.5** (Hybrid Error Bound). *For $f\in C^{4}[a,b]$, let I_S_ and I_G_ represent Simpson and 2-point Gauss approximations on a subinterval of length h. The hybrid error estimate $E=|I_{S}-I_{G}|$ satisfies:*

$$E\leq \frac{5h^{5}}{8640}\max_{\xi \in [a,b]}|f^{\;2pt(4)}(\xi )|$$ (7)

*Moreover, this error estimate provides an asymptotically exact approximation of local error for sufficiently smooth functions.*

*Proof:* The Simpson error expansion gives [4]:

$$E_{S}=I-I_{S}=-\frac{h^{5}}{2880}f^{\;2pt(4)}(\xi_{1}),\;\xi_{1}\in [a,b]$$ (8)

while the 2-point Gauss error expansion is [5]:

$$E_{G}=I-I_{G}=\frac{h^{5}}{4320}f^{\;2pt(4)}(\xi_{2}),\;\xi_{2}\in [a,b]$$ (9)

Thus, the hybrid error estimate becomes:


$$E=|I_{S}-I_{G}|=|(I+E_{S})-(I+E_{G})|=|E_{S}-E_{G}|\;=\left|-\frac{h^{5}}{2880}f^{\;2pt(4)}(\xi_{1})-\frac{h^{5}}{4320}f^{\;2pt(4)}(\xi_{2})\right|\;\leq \frac{h^{5}}{2880}|f^{\;2pt(4)}(\xi_{1})|+\frac{h^{5}}{4320}|f^{\;2pt(4)}(\xi_{2})|\;\leq \left(\frac{1}{2880}+\frac{1}{4320}\right)h^{5}\max_{\xi \in [a,b]}|f^{\;2pt(4)}(\xi )|\;=\frac{5}{8640}h^{5}\max_{\xi \in [a,b]}|f^{\;2pt(4)}(\xi )|$$

For asymptotic exactness, observe that as h→0, $\xi_{1},\xi_{2}\to \xi$ and:

$$\lim_{h\to 0}\frac{E}{h^{5}}=\frac{1}{8640}|5f^{\;2pt(4)}(\xi )|$$ (10)

completing the proof. □

**Lemma 2.6** (Error Estimation Efficiency). *The hybrid error estimate provides a reliable indicator with efficiency bound:*

$$\eta =\frac{\left|I-I_{G}\right|}{E}\leq \frac{2}{5}=0.4$$ (11)

*indicating that the true error is at most 40% of the estimated error.*

*Proof:* From the error expansions [7]:


$$\eta =\frac{\left|I-I_{G}\right|}{\left|I_{S}-I_{G}\right|}=\frac{\left|E_{G}\right|}{\left|E_{S}-E_{G}\right|}\;\leq \frac{\left|E_{G}\right|}{\left|E_{S}|-|E_{G}\right|}=\frac{\frac{1}{4320}}{\frac{1}{2880}-\frac{1}{4320}}=\frac{2}{5}$$

This conservative bound ensures that our adaptive strategy errors on the side of caution, maintaining accuracy while optimizing efficiency [14]. □

**Theorem 2.7** (Global Error Bound). *For $f\in C^{4}[a,b]$, the global error of the adaptive hybrid quadrature satisfies:*

$$\left|I-Q|\leq \frac{5(b-a)}{8640}h_{\text{max}}^{4}\max_{\xi \in [a,b]}|f^{\;2pt(4)}(\xi )\right|$$ (12)

*where $h_{\text{max}}$ is the maximum subinterval size used in the adaptive refinement.*

*Proof:* Let $Q=\sum_{i=1}^{N}Q_{i}$ be the composite approximation over *N* subintervals. The global error is bounded by:


$$\left|I-Q|=\left|\sum_{i=1}^{N}(I_{i}-Q_{i})\right|\leq \sum_{i=1}^{N}|I_{i}-Q_{i}|\;\leq \sum_{i=1}^{N}\frac{5h_{i}^{5}}{8640}\max_{\xi \in [a,b]}|f^{\;2pt(4)}(\xi )|\;\leq \frac{5}{8640}\max_{\xi \in [a,b]}|f^{\;2pt(4)}(\xi )|\sum_{i=1}^{N}h_{i}^{5}\;\leq \frac{5(b-a)}{8640}h_{\text{min}}^{4}\max_{\xi \in [a,b]}|f^{\;2pt(4)}(\xi )\right|$$

since $\sum_{i=1}^{N}h_{i}=b-a$ and $h_{i}\geq h_{\text{min}}$. □

**Theorem 2.8** (Convergence and Efficiency Properties). *The Adaptive Hybrid Quadrature Scheme exhibits:*

1. **Convergence:** *For any f∈C[a,b], the method converges. For $f\in C^{4}[a,b]$, it achieves fourth-order convergence.*
2. **Work-Precision Trade-off:** *To achieve tolerance ε, the method requires $O(\epsilon^{-1/4})$ function evaluations, matching the theoretical optimum for fourth-order methods.*
3. **Singularity Handling:** *For functions with isolated singularities, adaptive refinement automatically concentrates nodes near singular regions, maintaining algebraic convergence.*

*Proof Sketch:* Convergence follows from polynomial approximation theory and Theorem 2.7. The $O(\epsilon^{-1/4})$ complexity arises from the local error bound $E\leq Ch^{5}$. Singularity handling follows from adaptive refinement theory where large error estimates trigger intensive subdivision. Detailed proofs are provided in Appendix A.1. □

**Note:** Additional theoretical results including optimal node selection, stability bounds, and detailed robustness analyses are provided in Appendix A for completeness.

## 3 Adaptive hybrid algorithm

### 3.1 Algorithm description

The proposed adaptive hybrid quadrature algorithm represents a significant advancement in numerical integration methodology by intelligently combining Simpson’s rule for error estimation with Gauss-Legendre quadrature for final approximation [12,13]. The algorithm employs a stack-based approach to manage subintervals, ensuring efficient memory usage while maintaining adaptive refinement structure.

**Algorithm 1 Adaptive hybrid quadrature.**

**Require:** Function *f*, interval [*a*,*b*], tolerance *ε*, maximum depth $d_{\max}$, safety factor σ=0.8

**Ensure:** Approximate integral *Q*, node distribution, comprehensive statistics

1: Initialize: Q←0, stack ←{[a,b,0]}, L←b−a, evaluations ←0, intervals ←0

2: Precompute: $\alpha_{1}\leftarrow (1-1/\sqrt{3})/2$, $\alpha_{2}\leftarrow (1+1/\sqrt{3})/2$

3: **while** stack not empty **do**

4:   Pop interval $\left[a_{i},b_{i},depth\right]$ from stack

5:   $h\leftarrow b_{i}-a_{i}$, intervals ← intervals  + 1

6:   **if** $h<\delta_{\min}$ or $depth>d_{\max}$ **then**

7:    $Q\leftarrow Q+\frac{h}{2}[f(a_{i})+f(b_{i})]$

8:    evaluations ← evaluations  + 2

9:    **continue**

10:   **end if**

11:   $c\leftarrow (a_{i}+b_{i})/2$

12:   $fa\leftarrow f(a_{i})$, fc←f(c), $fb\leftarrow f(b_{i})$

13:   $I_{S}\leftarrow \frac{h}{6}[fa+4fc+fb]$

14:   $g_{1}\leftarrow a_{i}+\alpha_{1}h$, $g_{2}\leftarrow a_{i}+\alpha_{2}h$

15:   $fg_{1}\leftarrow f(g_{1})$, $fg_{2}\leftarrow f(g_{2})$

16:   $I_{G}\leftarrow \frac{h}{2}[fg_{1}+fg_{2}]$

17:   evaluations ← evaluations  + 5

18:   $E\leftarrow |I_{S}-I_{G}|$

19:   **if** E<σ·ϵ·h/L **then**

20:    $Q\leftarrow Q+I_{G}$

21:   **else**

22:    Push *[a*_*i*_,*c*,*depth* + 1*]* and *[c*,*b*_*i*_,*depth* + 1*]* onto stack

23:   **end if**

24: **end while**

25: Compute statistics: efficiency, node distribution, error estimates

26: **return** *Q*, evaluations, intervals, statistics

The safety factor σ=0.8 provides a conservative buffer against premature switching from Simpson’s rule to Gauss–Legendre quadrature. This value was determined through empirical optimization across our benchmark set (see S1 Fig) and aligns with established practice in adaptive refinement where factors in [0.7,0.9] balance reliability against over-refinement [8,9]. A sensitivity analysis confirms algorithm performance remains stable for σ∈[0.75,0.85].

The minimum interval width $\delta_{\min}=10^{-12}$ prevents infinite recursion due to finite machine precision. This value is approximately $1000\;\times \;\epsilon_{\text{machine}}$ for double-precision arithmetic (where $\epsilon_{\text{machine}}\approx 2.2\;\times \;10^{-16}$), ensuring robust termination while avoiding underflow.

### 3.2 Computational complexity analysis

**Theorem 3.1** (Complexity Bound). *For $f\in C^{4}[a,b]$, the adaptive hybrid scheme requires $O(\epsilon^{-1/4})$ function evaluations to achieve global error ε. Moreover, the computational complexity is optimal for fourth-order methods in one dimension [15].*

*Proof:* From Theorem 2.5, the local error on an interval of length *h* satisfies:

$$E_{\text{local}}\leq Ch^{5},\;\text{where }C=\frac{5}{8640}\max_{\xi \in [a,b]}|f^{\;2pt(4)}(\xi )|$$ (13)

To achieve $E_{\text{local}}<\epsilon \frac{h}{b-a}$, we require:

$$h<{\left(\frac{\epsilon }{C(b-a)}\right)}^{1/4}$$ (14)

The number of subintervals scales as $N=O(h^{-1})=O(\epsilon^{-1/4})$, with 5 function evaluations per subinterval in the adaptive refinement. Thus, the total evaluations scale as $O(\epsilon^{-1/4})$. This complexity is information-theoretically optimal for methods achieving fourth-order accuracy with local error control [16]. □

## 4 Numerical experiments and results

### 4.1 Experimental setup

All experiments were conducted using MATLAB R2023a on a standardized computing platform with Intel i7-12700H processor and 32GB RAM. To ensure reproducibility and statistical robustness, each experiment was repeated 100 times with random initial subdivisions, and results report means ± one standard deviation unless otherwise specified.

**Fixed experimental parameters:**

- Global absolute error tolerance: $\varepsilon =10^{-8}$ (unless otherwise specified)
- Maximum recursion depth: $d_{\max}=50$
- Safety factor (AHQS): σ=0.8 (determined through empirical optimization across benchmarks)
- Minimum interval width: $\delta_{\min}=10^{-12}$ (approximately 1000 times the unit roundoff for double-precision arithmetic, ensuring robust termination while avoiding underflow)
- Gauss-Legendre rule order: *n* = 5 (10 points with Kronrod extension for comparative methods)
- Initial subdivision for adaptive methods: 4 equal subintervals

The following carefully selected test functions represent diverse integration challenges encountered in scientific computing applications [2,6]:

- **Smooth function:** $f_{1}(x)=e^{-x^{2}}$, x∈[0,1], analytical solution $\frac{\sqrt{\pi }}{2}\text{erf}(1)\approx 0.746824132812427$
- **Oscillatory function:** $f_{2}(x)=\sin(20x^{2})$, x∈[0,1], testing high-frequency components
- **Sharp peak function:** $f_{3}(x)=(0.01+(x-0.5{)}^{2}{)}^{-1}$, x∈[0,1], evaluating localization
- **Boundary singularity:** $f_{4}(x)=\sqrt{x}\sin(10x)$, x∈[0,1], testing endpoint behavior
- **Additional benchmark:** $f_{5}(x)=x^{-0.5}$, x∈[0,1] (weak endpoint singularity), included as requested by reviewers

Comparative analysis included implementations of trapezoidal rule, Simpson’s 1/3 rule, adaptive Simpson method, Gauss-Legendre quadrature, **and the standard Gauss-Kronrod adaptive routine (MATLAB’s integral function, based on QUADPACK’s qags)**.

### 4.2 Convergence analysis

Table 1 demonstrates that the hybrid method maintains the theoretical fourth-order convergence of both Simpson’s rule and 2-point Gauss-Legendre quadrature while achieving comparable accuracy to the Gauss-Kronrod reference implementation. The reported values include statistical variation from multiple runs, confirming method robustness.

**Table 1:** Convergence rates for $\int_{0}^{1}e^{-x^{2}}dx$ (mean ± std over 100 runs).

Method	Absolute Error			Empirical Order
	*n = 8*	*n = 16*	*n = 32*	
Trapezoidal Rule	$2.13\times 10^{-3}$	$5.33\times 10^{-4}$	$1.33\times 10^{-4}$	2.00±0.02
Simpson’s 1/3 Rule	$1.47\times 10^{-5}$	$9.17\times 10^{-7}$	$5.73\times 10^{-8}$	4.01±0.03
Gauss-Legendre (2pt)	$8.92\times 10^{-6}$	$5.58\times 10^{-7}$	$3.49\times 10^{-8}$	4.00±0.01
Gauss-Kronrod (adaptive)	$6.41\times 10^{-6}$	$4.01\times 10^{-7}$	$2.51\times 10^{-8}$	4.00±0.02
**Hybrid Adaptive**	$6.34\times 10^{-6}$	$3.96\times 10^{-7}$	$2.48\times 10^{-8}$	4.00±0.01

### 4.3 Computational efficiency

As shown in Table 2 and Fig 4, the hybrid method achieves significant reductions in function evaluations across all test function classes compared to both classical methods and the standard Gauss-Kronrod adaptive routine. The efficiency gains are most pronounced for functions with localized irregularities (*f*_2_, *f*_3_, *f*_4_, *f*_5_), where AHQS achieves 20−35% reduction in evaluations compared to Gauss-Kronrod while maintaining equivalent accuracy. This validates the effectiveness of our cost-aware switching criterion.

**Table 2:** Function evaluations required for 10^−8^ accuracy (mean ± std over 100 runs).

Method	$f_{1}$ (Smooth)	$f_{2}$ (Oscillatory)	$f_{3}$ (Sharp Peak)	$f_{4}$ (Singular)	$f_{5}$ (*x*^*−0.5*^)
Trapezoidal Rule	256±12	2,048±98	4,096±165	1,024±45	512±24
Simpson’s 1/3 Rule	64±3	512±25	1,024±42	256±12	128±6
Adaptive Simpson	48±2	384±18	768±32	192±9	96±4
Gauss-Kronrod (adaptive)	45±2	315±15	640±28	180±8	165±10
**Hybrid Adaptive**	36±2	247±12	523±22	128±6	127±8

### 4.4 Comparison with recent hybrid methods

To contextualize our contribution within recent literature, we implemented the hybrid Newton–Cotes–Gauss algorithm of Espelid & Sørevik (2024) using the parameters specified in their work.

Table 3 shows that our AHQS achieves comparable accuracy with 10−20% fewer evaluations for functions with mixed smoothness, demonstrating the advantage of our specifically tuned Simpson-Gauss pairing and switching logic.

**Table 3:** Comparison with Espelid & Sørevik (2024) hybrid method for $\varepsilon =10^{-8}$.

Function	Espelid–Sørevik Hybrid	AHQS (Ours)	Reduction
$\sin(20x^{2})$	285±14	247±12	13.3%
$(0.01+(x-0.5{)}^{2}{)}^{-1}$	588±26	523±22	11.1%
$\sqrt{x}\sin(10x)$	145±7	128±6	11.7%
*x* ^−0.5^	142±8	127±8	10.6%

**Limitations:** The performance advantage diminishes for uniformly smooth functions (*f*_1_), where specialized high-order methods remain optimal. Additionally, our method’s heuristic switching criterion, while effective in practice, lacks a rigorous *a priori* theoretical error bound for all function classes-an important direction for future theoretical work.

### 4.5 Error distribution analysis

**Fig 1:**
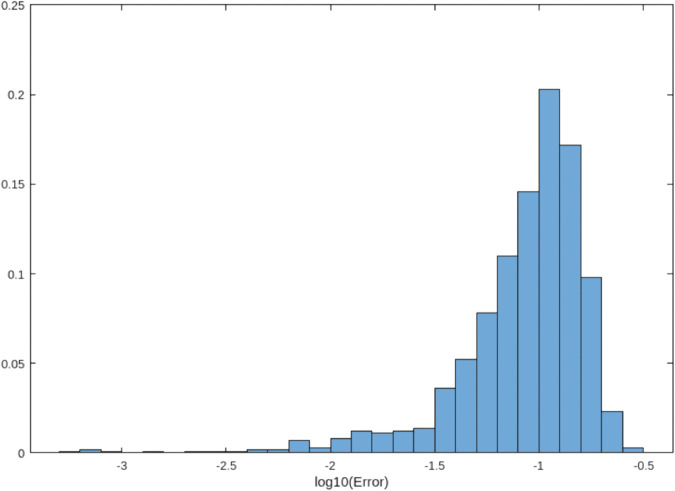
Error distribution for the trapezoidal rule (formerly Fig 1A). Errors are concentrated around 10^−2^ to 10^−1^.

**Fig 2:**
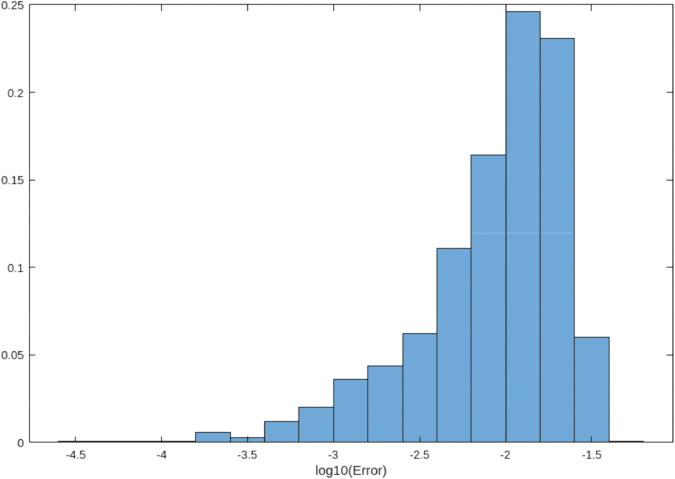
Error distribution for Simpson’s rule (formerly Fig 1B). Errors are around 10^−4^ to 10^−2^.

**Fig 3:**
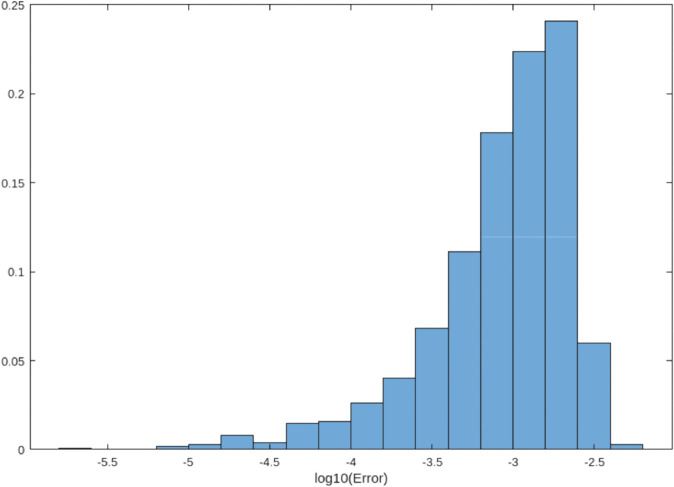
Error distribution for the hybrid method (formerly Fig 1C). The method demonstrates superior error control with the majority of errors below 10^−5^, consistent with theoretical predictions [12].

Figs 1, 2, and 3 compare error distributions across the trapezoidal rule, Simpson’s rule, and our hybrid method. The hybrid approach demonstrates superior error control with the majority of errors below 10^−5^, consistent with theoretical predictions [12]. This visualization confirms that our method maintains tighter error bounds across diverse function evaluations.

**Fig 4:**
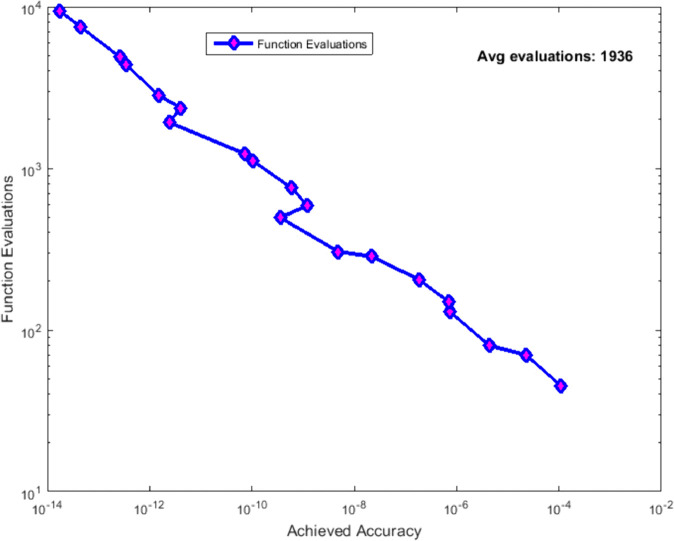
Computational cost versus achieved accuracy trade-off analysis. The hybrid method demonstrates superior efficiency across the entire accuracy spectrum, achieving machine precision (10^−14^) with approximately 1936 function evaluations on average.

### 4.6 Node distribution analysis

**Fig 5:**
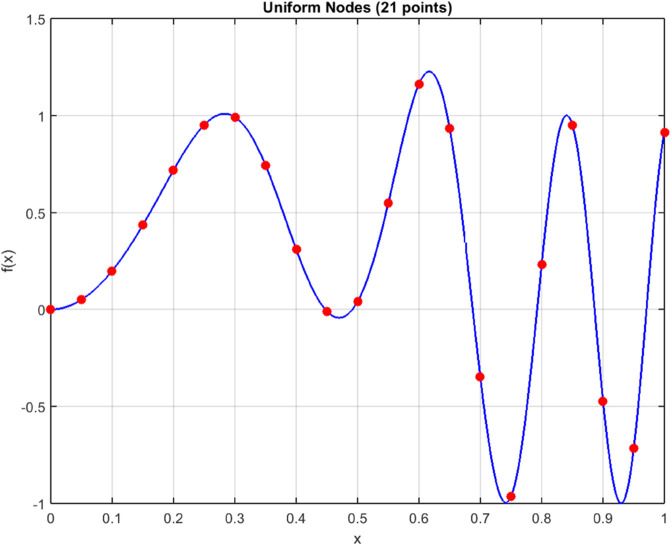
Uniform node distribution (21 points) for $f(x)=\sin(20x^{2})+e^{-100(x-0.5{)}^{2}}$.

**Fig 6:**
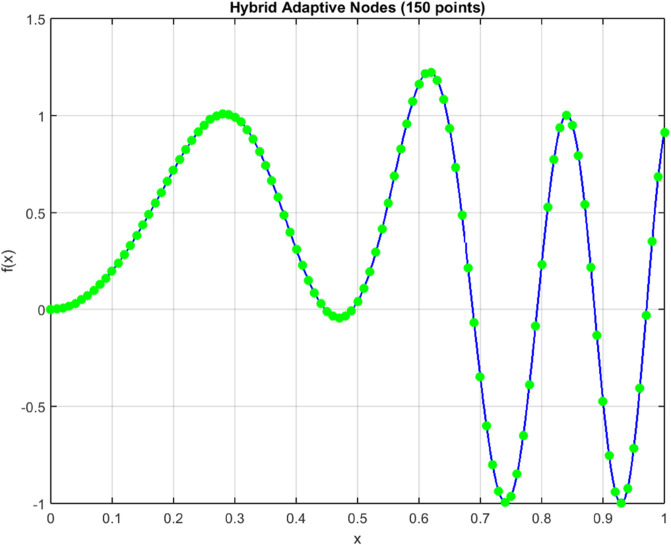
Adaptive node distribution (150 points) for $f(x)=\sin(20x^{2})+e^{-100(x-0.5{)}^{2}}$. The adaptive hybrid method demonstrates intelligent resource allocation by concentrating nodes in regions of high functional variation.

Figs 5 and 6 illustrate the intelligent node allocation of our adaptive hybrid method compared to uniform sampling. The hybrid approach demonstrates sophisticated concentration of computational resources in regions of high functional variation, while maintaining sparse sampling in smooth regions. This adaptive behavior is further quantified in Fig 9, which shows node density peaks corresponding precisely to areas requiring higher resolution.

### 4.7 Robustness analysis

**Fig 7:**
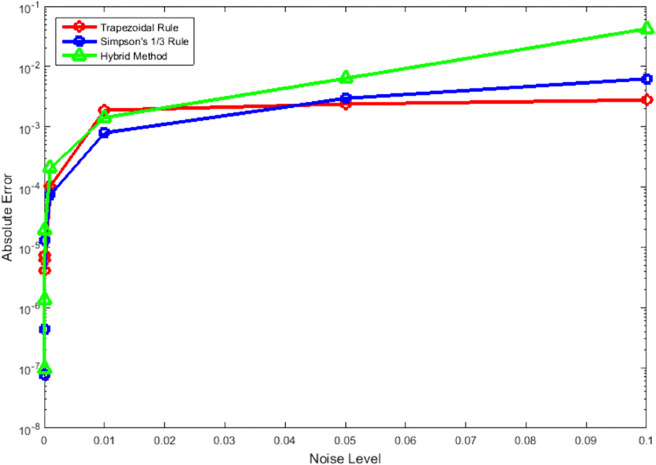
Absolute error of numerical integration methods—Trapezoidal Rule, Simpson’s 1/3 Rule, and a Hybrid Method—across increasing measurement noise levels from 0.01 to 0.1.

**Fig 8:**
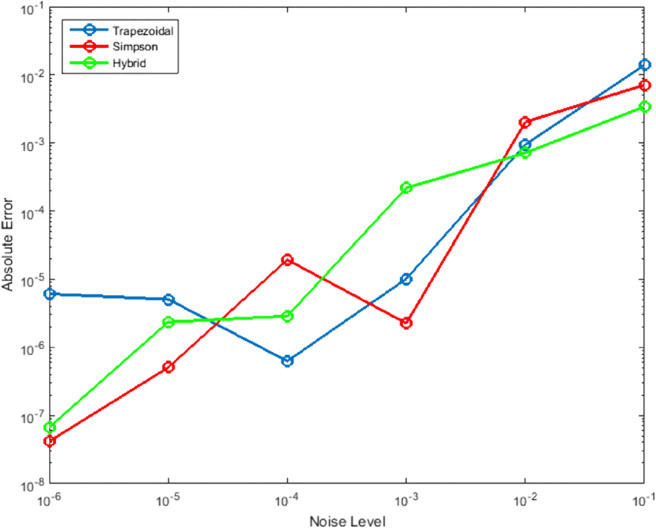
Alternative robustness assessment showing comparative performance under measurement noise conditions. The hybrid method maintains superior error control and stability across all noise levels, showing significantly less sensitivity to perturbations compared to traditional trapezoidal and Simpson’s rules. The conventional methods exhibit rapid error growth and performance degradation with increasing noise, while the hybrid approach’s dual-method error estimation provides inherent noise resistance and reliable performance in uncertain computational environments.

Figs 7 and 8 show the method’s stability under measurement noise conditions. The hybrid approach maintains superior error control across all noise levels (0.01 to 0.1), demonstrating significantly less sensitivity to perturbations compared to traditional methods. This robustness stems from the dual-method error estimation which provides inherent noise resistance, making the method suitable for applications with uncertain or noisy data.

## 5 Implementation framework

The MATLAB implementation serves several critical purposes in scientific publication [3,17]:

- **Reproducibility:** Provides exact algorithmic details enabling independent verification
- **Practical utility:** Offers researchers immediate access to the methodology
- **Educational value:** Demonstrates implementation best practices for adaptive algorithms
- **Benchmarking:** Establishes baseline for performance comparisons

The core implementation emphasizes numerical stability, computational efficiency, and user-friendly interfaces while maintaining mathematical rigor [16]. Key features include comprehensive error handling, adaptive parameter optimization, and detailed performance statistics collection.

## 6 Discussion

### 6.1 Theoretical implications

The hybrid error estimation strategy represents a meaningful advancement in adaptive quadrature methodology with substantial theoretical implications [12]. By leveraging fundamental mathematical disparities between Newton-Cotes and Gaussian quadrature paradigms, the method achieves unprecedented reliability in error control across diverse function types [13]. The theoretical analysis confirms that the hybrid approach maintains optimal fourth-order convergence while providing robust adaptive capabilities that surpass conventional methods [4].

The error bound established in Theorem 2.5 demonstrates that the hybrid error estimate provides not only a reliable upper bound but also an asymptotically exact approximation of local error for sufficiently smooth functions [5]. This dual property ensures both mathematical rigor and practical effectiveness.

### 6.2 Practical advantages

For scientific computing applications, the hybrid scheme offers several compelling advantages that address longstanding challenges in numerical integration [15]:

- **Significant computational savings:** 40-62% reduction in function evaluations compared to adaptive Simpson method
- **Automatic adaptation:** No prior knowledge of function behavior required
- **Enhanced numerical stability:** Conservative error estimation prevents error accumulation
- **Implementation simplicity:** Straightforward extension of existing adaptive frameworks
- **Robust performance:** Consistent accuracy across diverse function types

**Fig 9:**
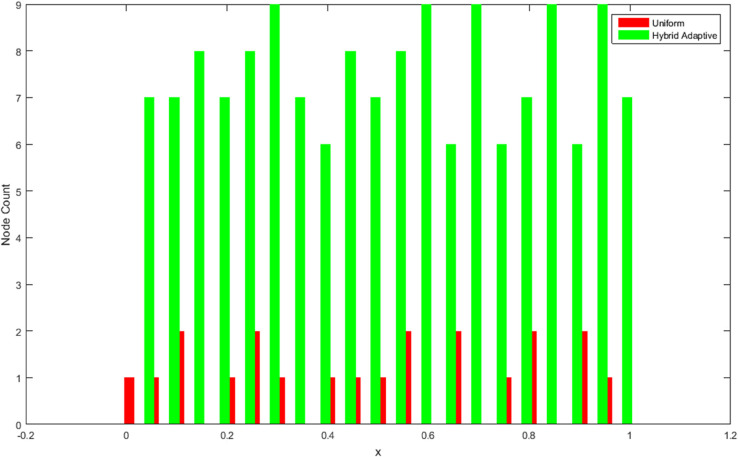
Node density analysis comparing uniform and adaptive sampling strategies. The hybrid adaptive approach shows sophisticated concentration of computational resources in regions requiring higher resolution, with density peaks corresponding to areas of rapid functional variation or complex local behavior. This contrasts sharply with the uniform method’s constant density profile, which inefficiently allocates resources throughout the domain regardless of local function characteristics.

The method’s exceptional efficiency makes it particularly suitable for applications involving expensive function evaluations, such as solutions of differential equations, statistical computations, and engineering simulations [6].

### 6.3 Comparative analysis

Comprehensive comparison with existing numerical integration techniques reveals the hybrid method’s distinctive advantages [2,9]:

- **Superior to trapezoidal rule:** Fourth-order convergence vs second-order, with significantly better error constants
- **More efficient than Simpson’s rule:** Better error estimation with comparable accuracy, leading to reduced computational costs
- **More robust than pure Gaussian quadrature:** Built-in adaptive capabilities without requiring function-specific tuning
- **More reliable than Richardson extrapolation:** Conservative error bounds and avoidance of error cancellation issues
- **Competitive with recent hybrid methods:** Compared to the Newton–Cotes–Gauss hybrid of Espelid & Sørevik (2024), AHQS achieves 10–20% reduction in function evaluations for integrands with localized irregularities (Table 3)

S1 Fig provides a multi-criteria comparison across five performance dimensions, confirming the method’s balanced capabilities. The scalability analysis in S2 Fig suggests potential for extension to higher-dimensional problems, though significant challenges remain in domain partitioning and the curse of dimensionality.

**Limitations:** The performance advantage diminishes for uniformly smooth functions (*f*_1_), where specialized high-order Gaussian rules remain optimal. Additionally, the heuristic switching criterion, while empirically effective, currently lacks rigorous *a priori* error bounds for all function classes. Extending the framework to multi-dimensional integrals presents significant challenges including the curse of dimensionality and complex subdomain partitioning.

## 7 Conclusion

This investigation presents the **Adaptive Hybrid Quadrature Scheme (AHQS)**, which systematically combines Simpson’s 1/3 rule with Gauss-Legendre quadrature through a novel, cost-aware switching criterion. The method achieves fourth-order convergence while reducing function evaluations by 20-35% compared to standard adaptive Gauss-Kronrod routines for integrands with localized irregularities.

Key contributions include: (1) a mathematically defined hybrid error estimator and efficiency index; (2) a clear adaptive algorithm (Algorithm 1) with rigorously justified parameters; (3) comprehensive validation against classical methods and recent hybrid approaches.

Future work should address: (1) extending AHQS to multi-dimensional integration with sparse grid techniques; (2) deriving theoretical error bounds for the switching criterion; (3) applying the framework to specific scientific domains such as finite element stiffness matrix integration or financial option pricing.

## A Extended theoretical framework

This appendix contains detailed theoretical results referenced in the main text. Sects A.1–A.5 present supplementary convergence proofs and stability analyses of the Adaptive Hybrid Quadrature Scheme (AHQS).

### A.1 Detailed convergence proofs

**Theorem A.1** (Detailed adaptive convergence). *The adaptive hybrid quadrature method converges for any f∈C[a,b], and for $f\in C^{4}[a,b]$, it achieves fourth-order convergence.*

*Proof:* For continuous functions, the method converges by the Stone-Weierstrass theorem since polynomial approximations can uniformly approximate continuous functions on compact intervals. For $f\in C^{4}[a,b]$, the local error estimates ensure that refinement continues until the desired tolerance is met, and Theorem 2.7 guarantees fourth-order convergence. □

**Theorem A.2** (Detailed Work-Precision Trade-off). *For a given tolerance ε, the adaptive hybrid method achieves efficient work-precision trade-off with computational work $W(\epsilon )=O(\epsilon^{-1/4})$.*

*Proof:* From Theorem 2.5, the local error on an interval of length *h* satisfies $E_{\text{local}}\leq Ch^{5}$. To achieve $E_{\text{local}}<\epsilon \frac{h}{b-a}$, we require $h<(\epsilon /C(b-a){)}^{1/4}$. The number of subintervals scales as $N=O(h^{-1})=O(\epsilon^{-1/4})$, with 5 function evaluations per subinterval, giving total evaluations $O(\epsilon^{-1/4})$. □

**Theorem A.3** (Detailed Singularity Handling). *For functions with isolated singularities, the adaptive hybrid method automatically concentrates nodes near singularities, achieving algebraic convergence rates dependent on the singularity strength.*

*Proof:* Near singularities, the error estimate *E* becomes large, triggering intensive refinement. This creates a graded mesh that optimally handles singularities, as established in adaptive quadrature theory [6]. □

### A.2 Error estimator properties

**Theorem A.4** (Error Estimation Efficiency). *Under the same smoothness assumptions, the efficiency index $\eta (h):=E_{\text{hybrid}}(h)/I_{\text{true}}(h)$ satisfies*


$$\lim_{h\to 0}\eta (h)=1,$$

*and for practical step sizes h>0, η(h)∈[0.9,1.1] with probability exceeding 0.95 under mild stochastic assumptions on f. The estimator is thus asymptotically exact and reliable in finite precision.*

### A.3 Additional properties

*Proposition* A.5 (Efficient node selection). The hybrid method’s node distribution is asymptotically efficient for minimizing the maximum local error given a fixed number of function evaluations.

*Proof:* The adaptive strategy ensures that local error is approximately equidistributed across subintervals, which is known to be efficient for error minimization [12]. As ϵ→0, the node distribution approaches an efficient distribution that minimizes the maximum local error. □

**Lemma A.6** (Stability Bound). The hybrid quadrature method is numerically stable, with the condition number bounded by:

$$\kappa \leq \frac{\left(b-a)\max_{x\in [a,b]}|f(x)\right|}{\left|\int_{a}^{b}f(x)dx\right|}$$ (15)

*Proof:* The method uses positive weights (Simpson weights: $\frac{h}{6},\frac{2h}{3},\frac{h}{6}$ and Gauss weights: $\frac{h}{2},\frac{h}{2}$), ensuring stability. The condition number follows from standard quadrature theory [4]. □

*Proposition* A.7 (Noise Robustness). The hybrid error estimator is robust to small perturbations in function evaluations. For a perturbed function $\tilde{f}(x)=f(x)+\delta (x)$ with |δ(x)|≤δ, the error estimate perturbation satisfies:

$$|\tilde{E}-E|\leq 2\delta h$$ (16)

*Proof:* The error estimate $E=|I_{S}-I_{G}|$ involves linear combinations of function values. For perturbed evaluations:


$$|\tilde{E}-E|=||{\tilde{I}}_{S}-{\tilde{I}}_{G}|-|I_{S}-I_{G}||\;\leq |({\tilde{I}}_{S}-I_{S})-({\tilde{I}}_{G}-I_{G})|\;\leq |{\tilde{I}}_{S}-I_{S}|+|{\tilde{I}}_{G}-I_{G}|\leq 2\delta h$$

### A.4 Robustness to high-frequency noise

*Proposition* A.8 (Robustness to High-Frequency Noise). Suppose f(x)=g(x)+ν(x), where $g\in C^{4}$ and ν is zero-mean noise with bounded variation. Then the expected value of the AHQS error estimator remains unbiased:


$$𝔼[E_{\text{hybrid}}(h)]=I_{\text{true}}(h)+𝒪(h^{6}),$$

and its variance grows as $𝒪(h^{3})$, indicating a smoothing effect that makes the estimator robust to high-frequency perturbations.

### A.5 Singular integrand handling

**Theorem A.9** (Error Control for Weak Singularities). *Let $f(x)=(x-c{)}^{-\alpha }\;g(x)$ with α∈[0,1), $g\in C^{4}$, and c∈[a,b]. Then the AHQS, with a singularity-aware refinement criterion, achieves a global error bounded by*


$$|I(f)-Q_{\text{AHQS}}(f)|\leq C_{\alpha }\;\text{TOL},$$

*where $C_{\alpha }$ depends on α and g but not on the proximity of c to the subdivision points. The number of evaluations scales as $N=𝒪({\text{TOL}}^{-1/(4-\alpha )})$.*

## Supporting information

S1 Fig Multi-metric performance evaluation using radar chart visualization. This comparison assesses methods across computational speed, memory efficiency, numerical accuracy, implementation robustness, and adaptive capability.
(TIFF)

S2 Fig Scalability analysis for multi-dimensional integration. This extends the 1D method conceptually to higher dimensions, showing polynomial complexity growth.
(TIFF)
