# Supplementary material for: An adaptive hybrid quadrature scheme: Combining Simpson’s rule and Gaussian quadrature for enhanced numerical integration
Source: PLoS One. 2026 Feb 23;21(2):e0335582. doi: 10.1371/journal.pone.0335582 (PMC12928599; doi:10.1371/journal.pone.0335582)
Supplement: Attachment — Submitted filename: PLOS_ONE.pdf [file pone.0335582.s003.pdf]

Referee Report

Authors: Abadi Abraha Asgedom, Yohannes Yirga Kefela
Manuscript Title: *An Adaptive Hybrid Quadrature Scheme: Combining Simpson's Rule and Gaussian Quadrature*

The manuscript investigates an adaptive quadrature method that combines Simpson's rule with the 2-point Gauss–Legendre formula, using their discrepancy as a local error estimator. The topic is relevant in numerical integration, the exposition is generally clear, and the idea is intuitive. The numerical tests are rich, and the implementation is transparent. The manuscript has potential, but several aspects require revision to improve clarity, conciseness, and scientific framing.

The work's strengths include its accessible presentation, the clarity of the algorithm, and the breadth of numerical tests. The hybrid error estimator is reasonable, and the authors' effort in providing implementation details is appreciated.

However, some parts of the manuscript are significantly longer than necessary, particularly in the introduction and theoretical background, where several results restate classical material. Certain claims (especially regarding optimality or performance on rapidly varying functions) appear too strong in their current form. A comparison with standard Gauss–Kronrod adaptive routines would considerably strengthen the numerical validation. Some figures seem non-essential for a 1D quadrature method. Minor linguistic improvements would also enhance readability.

The following revisions are suggested to help the authors refine the manuscript in a focused and effective manner.

1. Consider reducing the introduction by approximately 20–30%. The historical discussion of Newton–Cotes formulas, Gaussian quadrature, and general background material can be substantially shortened so that the motivation and contribution emerge more clearly.

2. The theoretical section could be consolidated. Theorems 2.3, 2.4, and 2.5 may be merged or summarized, while results such as Theorems 2.7, 2.8, 2.10, and 2.11 could be moved to an Appendix to streamline the exposition.

3. Some strong statements should be moderated. Claims such as "optimal work-precision trade-off", "optimal node distribution", or "unprecedented robustness" may require either further evidence or a more cautious formulation (e.g., "numerical evidence suggests...", "the method shows promising behavior...").

4. The manuscript emphasizes the ability of the proposed error estimator to detect regions where the function exhibits rapid local variation. To provide readers with a broader perspective, it may be useful to mention global reconstruction strategies that remain accurate even in the presence of strong but smooth functional variations. In particular, several mock-Chebyshev constrained least-squares methods have shown robustness against the Runge phenomenon and stability for rapidly varying functions, thanks to their global approximation structure.

The authors may therefore wish to mention the following related works:

- *An adaptive algorithm for determining the optimal degree of regression in constrained mock-Chebyshev least squares quadrature* (Dell'Accio, Di Tommaso, Francomano & Nudo, *Dolomites Research Notes on Approximation*, 2022, 15(4), pp. 35–44);

- *Constrained mock-Chebyshev least squares quadrature* (Dell'Accio, Di Tommaso & Nudo, *Applied Mathematics Letters*, 2022, 134, Article 108328);
- *An interpolation–regression approach for function approximation on the disk and its application to cubature formulas* (Dell'Accio, Marcellán & Nudo, *Advances in Computational Mathematics*, 2025, 51:53);
- *A quadrature formula on triangular domains via an interpolation–regression approach* (Dell'Accio, Marcellán & Nudo, *Applied Mathematics Letters*, 2025, 163, 109414).

These works illustrate how hybrid approximation–quadrature techniques can be generalized from one dimension to higher-dimensional domains, including disks and triangles. Although the underlying philosophy differs (global reconstruction vs. local adaptivity), both approaches aim to improve stability and accuracy in the presence of rapidly varying functions, and mentioning them would help readers situate the present contribution within the broader landscape of modern hybrid integration methods.

5. A benchmark against a standard Gauss–Kronrod routine (e.g., QUADPACK's `qags` or `qag`) on at least one test function, with the same tolerance, would significantly strengthen the numerical section.

6. Some figures may be simplified or removed. In particular, the radar chart and the high-dimensional scalability plot appear tangential to the core contribution and could be moved to supplementary material.

7. Minor stylistic and linguistic adjustments would improve readability, especially in the discussion where some expressions are repeated.

8. The conclusion could be shortened and made more focused by identifying two or three realistic directions for future work.

Final Remark. The manuscript contains an interesting idea and promising numerical evidence. Addressing the points above would considerably improve clarity, conciseness, and scientific framing, helping the contribution become more accessible and convincing for the readership.
